# Supplementary material for: Pathophysiological Significance of Neutrophilic Transfer RNA-Derived Small RNAs in Asymptomatic Moyamoya Disease
Source: Cells. 2021 May 1;10(5):1086. doi: 10.3390/cells10051086 (PMC8147334; doi:10.3390/cells10051086)

2D Structure	Local AU	Position

```
126                          139
            8mer-1a
5'- a g t c g c T C A C A G G a -3' utr
                | | | | | | |
3'- g u u u u c A G U G U C C g -5' tRF
   16 15  14 13    7 6 5 4 3 2
 3' Pairing           Seed
```

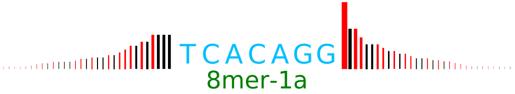

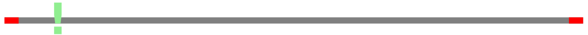

2D Structure	Local AU	Position

```
411                          424
            8mer-1a
5'- a t t t g t T C A C A G G a -3' utr
                | | | | | | |
3'- g u u u u c A G U G U C C g -5' tRF
   16 15  14 13    7 6 5 4 3 2
 3' Pairing           Seed
```

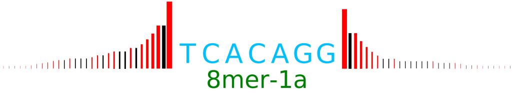

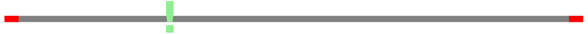

Supplement: Supplementary file 1 [file cells-10-01086-s001.zip › cells-1149161 3rd supplementary materials/Data Supplements/Data Supplement V.zip!/Data supplement 6-mRNAs targets of tsRNAs/tsRNA-Arg-CCT-008/Diagrams/tRF-Arg-CCT-008_vs_ENST00000273261(LRIG1).pdf]
